# Replication of *KCNJ11* (p.E23K) and *ABCC8* (p.S1369A) Association in Russian Diabetes Mellitus 2 Type Cohort and Meta-Analysis

**DOI:** 10.1371/journal.pone.0124662

**Published:** 2015-05-08

**Authors:** Ekaterina Alekseevna Sokolova, Irina Arkadievna Bondar, Olesya Yurievna Shabelnikova, Olga Vladimirovna Pyankova, Maxim Leonidovich Filipenko

**Affiliations:** 1 Laboratory of Pharmacogenomics, Institute of Chemical Biology and Fundamental Medicine, Siberian Division, Russian Academy of Sciences, Novosibirsk, Russia; 2 Novosibirsk State University, Novosibirsk, Russia; 3 Novosibirsk State Regional Hospital, Regional Diabetes center, Novosibirsk, Russia; 4 Novosibirsk State Medical University, Novosibirsk, Russia; 5 Kazan Federal University, Kazan, Russia; Odense University hospital, DENMARK

## Abstract

The genes *ABCC8* and *KCNJ11* have received intense focus in type 2 diabetes mellitus (T2DM) research over the past two decades. It has been hypothesized that the p.E23K (*KCNJ11*) mutation in the 11p15.1 region may play an important role in the development of T2DM. In 2009, Hamming et al. found that the p.1369A (*ABCC8*) variant may be a causal factor in the disease; therefore, in this study we performed a meta-analysis to evaluate the association between these single nucleotide polymorphisms (SNPs), including our original data on the Siberian population (1384 T2DM and 414 controls). We found rs5219 and rs757110 were not associated with T2DM in this population, and that there was linkage disequilibrium in Siberians (D’=0.766, r^2^= 0.5633). In addition, the haplotype rs757110[T]-rs5219[C] (p.23K/p.S1369) was associated with T2DM (OR = 1.52, 95% CI: 1.04-2.24). We included 44 original studies published by June 2014 in a meta-analysis of the p.E23K association with T2DM. The total OR was 1.14 (95% CI: 1.11-1.17) for p.E23K for a total sample size of 137,298. For p.S1369A, a meta-analysis was conducted on a total of 10 studies with a total sample size of 14,136 and pooled OR of 1.14 [95% CI (1.08-1.19); p = 2 x 10^-6^]. Our calculations identified causal genetic variation within the *ABCC8*/*KCNJ11 *region for T2DM with an OR of approximately 1.15 in Caucasians and Asians. Moreover, the OR value was not dependent on the frequency of p.E23K or p.S1369A in the populations.

## Introduction

Type 2 diabetes mellitus (T2DM) is a pandemic that affects 6% of the adult population in developed countries [1]. Both genetic and environmental factors play a role in the development of T2DM. In particular, *ABCC8*(ATP-binding cassette, sub-family C (CFTR/MRP), member 8) and *KCNJ11*(potassium channel, inwardly rectifying subfamily J, member 11) have been the focus of T2DM research over the past two decades due to their possible role in the pathogenesis. These genes are located4.5 kb apart on chromosome 11p15.1 and encode the human Kir6.2 (*KCNJ11*) and SUR1 (*ABCC8*) subunits of plasma membrane potassium (K) ATP channels expressed in pancreatic β-cells. The promoters of both genes have been cloned [2], and polymorphisms in these promoters can lead to aberrant expression of K ATP channels, which can consequently disrupt the normal stoichiometry (4 Kir6:4 SUR1) of the two subunits that is essential for proper channel function [3]. It is well-known that mutations in these genes cause the autosomal recessive disorder familial persistent hyperinsulinemic hypoglycemia of infancy[4,5]. In 2002,Schwanstecher et al. provided evidence that ap.E23K polymorphism in *KCNJ11*(SNP rs5219) alters protein function by inducing the spontaneous overactivation of pancreatic β-cells. This results in an increase in the threshold ATP concentration required for insulin release [6]. SNP rs5219 has been shown to be associated with T2DM in many populations of Europe and East Asia, but not in Ashkenasi Jewish [7], Mongolian [8], or Indian [9,10] populations. It has been hypothesized that the rs5219 (p.E23K) variation in the 11p15.1 region may play an important role in the development of T2DM, thus making it a popular marker to assess in*KCNJ11*.However, to date the influence of *ABCC8* SNPs on the susceptibility to T2DM has not been well-characterized. Additional SNPs within *ABCC8*have been studied for predisposition toT2DM(e.g., exon 16: c.2117-3C>T, exon 18: p.T759T (ACC->ACT, rs1801261), and exon 33: p.S1369A).It is difficult to generalize the results between the different association studies on the SNPs of *ABCC8*.In 2009, Hamming et al. found that the p.K23/p.1369A haplotype that resulted from a direct effect of the *ABCC8*p.1369A risk allele led to a decrease in ATP inhibition, which was likely due to mild increases in intrinsic K ATP channel MgATPase activity. Moreover, a strong linkage disequilibrium in the 11p15.1 region has been observed in European populations (r^2^ = 0.98) [11]. It would thus be necessary to genotype both polymorphisms in populations in which p.E23K and p.S1369A mutations are present at high frequency. Earlier association studies of these polymorphisms in *KCNJ11* and *ABCC8* were only conducted in the regions of Russia near Europe [12], but not Siberia. Therefore, the first aim of this study was to explore associations between T2DM and two SNPs, *KCNJ11* (p.E23K) and *ABCC8* (p.S1369A), in a Siberian population. To the best of our knowledge, this is the largest study reported to date on the association of these SNPs in Russians with T2DM.Despite the clear functional role that these two genes may have in the pathogenesis of T2DM as well as several association and meta-analysis studies, the generalizing conclusion for all races have not been done. All previous meta-analyses of p.E23K and p.S1369A included different groups of selected studies, and some studies were not included. To date, there has been no summary analysis of all previous results. Therefore, the second aim of this study was to perform a meta-analysis of these SNPs based on data taken from all available previous studies as well as our original data.

## Materials and Methods

### Participants

The study population was comprised of 1384 individuals with T2DM (Female = 78%, mean age ± SD of 59.7±8.6 y, mean BMI = 33.6 ± 6.5 kg/m^2^) and the control group included 414 healthy individuals (Female = 57%, mean age ± SD of 32.7±10.6 y, mean BMI = 23.6 ± 4.0 kg/m^2^). Diabetic and control individuals were recruited at the diabetes center at the Novosibirsk Regional State Hospital. All individuals participating in the study were members of the European Russian ethnic group. The protocol (#52) was approved by The Local Ethics Committee of the Novosibirsk State Medical University on March 19^th^, 2013. All participants signed a written informed consent. All consecutive patients were deemed eligible pending signed informed consent and meeting inclusion/exclusion criteria. A control group was included that consisted of volunteers who were 18 years older with normal fasting and normal 2-h oral glucose tolerance test (OGTT).T2DM was defined according to the WHO 1999 criteria [13]. A clinical examination was performed that included an interview, anthropometric measurements, and blood collection. Distributions of the primary phenotypes are listed in Table 1.

**Table 1:** Demographic summary of European Russian participants.

	Diabetes mellitus 2 type		Control	
	Mean ± SD	Median	Mean ± SD	Median
Age (years)	59.7 ± 8.6	60.0	32.7±10.6	31.0
BMI (kg/m^2^)	33.6 ± 6.5	32.9	23.6 ± 4.0	23.1
HbA_1c_(mmol/mol)	71.6 ± 0.6	69.4	-	-
C-peptide (pmol/l)	674.2 ± 414.4	619.0	-	-
Women (%)	78%		57%	
Subjects, *n*	1384		414	

### Genotyping

DNA was isolated from venous blood using a standard procedure. Briefly, samples were collected and the blood was separated and lysed. Protein was then hydrolyzed with proteinase K and the DNA was extracted using phenol-chloroform followed by precipitation with ethanol. Genotyping of the SNPs was performed by real-time PCR using the following TaqMan probes(rs5219: forward primer 5’-ATACGTGCTGACACGCCTG-3’, reverse primer 5’-TGCCTTTCTTGGACACAAAGC-3’, 5’-R6G-ACCCTGCCGAGCCCA-BHQ-3’, 5’-FAM-ACCCTGCCAAGCCCA-BHQ-3’; rs757110: forward primer 5’-CTACGACAGCTCCCTGAAGC-3’, reverse primer 5’-TGACTGCGAAGCCATCC-3’, 5’-FAM-CCCTCATCTCCCCTGGACA-BHQ-3’, 5’-R6G-CCCTCATCGCCCCTGG-BHQ-3’).The PCR mixture contained DNA (40–100 ng), each primer (300 nM), TaqMan probes conjugated with FAM or R6G (100–200 nM each), dNTPs (200 μM), amplification buffer, and Taqpolymerase (0.5 U/reaction) in a total volume of 25 μL. Amplification was performed using aCFX96 cycler (Bio-Rad, USA) under the following conditions: initial denaturation for 3 min at 96ºC followed by40 cycles of denaturation at 96ºC for 10 s and annealing of primers and subsequent elongation at 60ºC for 40 s. The call rate for both SNPs was 100%.Case and control samples were plated together. We also included 10% duplicate pairs for both case and control samples. The concordance was >99%.

### Statistical data analysis

The Hardy-Weinberg Equilibrium was evaluated using an exact test of Hardy-Weinberg Equilibrium for 2-Allele markers in the R package “genetics”. Possible associations between SNPs and disease development were found using logistic regression analysis adjusted for age and gender, as implemented in the “glm” function of the R package for statistical analysis (www.r-project.org).Meta-analysis and estimated heterogeneity were carried out using the ‘rmeta’ package for R (http://cran.r-project.org/web/packages/rmeta/rmeta.pdf). Pooled odds ratios (ORs) were computed by the fixed-effect model for data combined under no heterogeneity between studies. If there was significant heterogeneity between studies, then the random effects model was applied for combined data. Haplotype analysis was carried out using the ‘haplo.stats’ package for R (http://cran.r-project.org/web/packages/haplo.stats/haplo.stats.pdf).Results were considered statistically significant for all statistical calculations if *P*< 0.05.The power of the study was calculated using software available online (http://pngu.mgh.harvard.edu/~purcell/gpc/cc2.html).

## Results

### Association of SNPs with T2DM

In this study we assessed SNP genotypes [rs5219 (p.E23K) and rs757110 (p.S1369A)] and found that the distribution of both SNPs corresponded to a Hardy-Weinberg equilibrium (HWE) in T2DM patients and control groups (Table 2). The frequencies of occurrence of the T allele of rs5219 in *KCNJ11* were 0.63 and 0.62 in control and case groups, respectively. The frequencies of occurrence of the G allele of rs757110 in *ABCC8* were 0.61 and 0.62 in control and case groups, respectively. Associations between the T2DM and genotypes of SNPs were estimated using logistic regression analysis adjusted for age and gender for three inheritance models: additive, dominant, and recessive. We found that neither rs5219 nor rs757110 were associated with T2DM in this patient population.

**Table 2:** Odds Ratio for Three Genetic Models for SNPs: rs5219 and rs757110.

Gene (SNP)	Control (total = 414)	T2DM (total = 1384)	OR (95% CI) co-dominant model, p-value, AIC	OR (95% CI) additive model, p-value, AIC	OR (95% CI) dominant model, p-value, AIC	OR (95% CI) recessive model, p-value, AIC
KCNJ11 (rs5219)	CC/CT/TT 158/204/52HWE = 0.29 RAF = 0.37	CC/CT/TT 535/656/193HWE = 0.77 RAF = 0.38	СС: referenceCT: 0.95 [0.75–1.20] p = 0.67TT: 1.10 [0.77–1.56] p = 0.61AIC = 1945.6	1.02 [0.87–1.20] p = 0.81AIC = 1944.3	0.98 [0.78–1.23] p = 0.86AIC = 1944.3	1.13 [0.81–1.57] p = 0.47AIC = 1943.8
ABCC8 (rs757110)	TT/TG/GG 160/189/65HWE = 0.47RAF = 0.39	TT/TG/GG 526/651/207HWE = 0.82RAF = 0.38	TT: referenceTG: 1.08 [0.78–1.49] p = 0.63 GG: 1.03 [0.74–1.44] p = 0.85AIC = 1946.1	1.00 [0.85–1.17] p = 0.98AIC = 1944.3	1.03 [0.82–1.29] p = 0.81AIC = 1944.3	0.94 [0.75–1.18] p = 0.71AIC = 1944.2

AIC—Akaike Information Criterion, lower the AIC value better the model.Abbreviations: HWE—p-value of Hardy-Weinberg equilibrium, RAF—risk allele freaquency (T for *KCNJ11*(rs5219) and G for *ABCC8* (rs757110)).

### Meta-analysis of rs5219 of *KCNJ11*

#### Study selection and characteristics of included studies

All published studies that evaluated the association between rs5219 of *KCNJ11* (p.E23K) and T2DM or NIDDM were collected through a PubMed search of studies published before June 2014 using the search terms “KCNJ11”, “polymorphism”, “T2DM”, and “rs5219” in different combinations. Studies included in our meta-analysis met the following criteria: 1) conducted as a case-control design; 2) evaluated the association of rs5219 and T2DM; 3) written in English or included an abstract in English with sufficient information; 4) reported sufficient data for an odds ratios (OR) calculation; and 5) reported sufficient data for obeying HWE. We also performed a manual search of references for potentially relevant articles, and missing information was requested from article authors. If a reply was not forthcoming, then the study was excluded from the meta-analysis. Allele frequencies were calculated from the corresponding genotype distributions when not provided: [Fr._p.23K_ = (2N_KK_+N_EK_)/2*(N_EE_+N_EK_+N_KK_)]. In addition, we found several meta-analyses that had been previously published. Available information on the study design, race or ethnicity of participants, first author, published year, reference, sample size of case and control, OR, 95% confidence intervals, and rare allele frequency in the control group are shown in Table 3.

**Table 3:** Characteristics studies of association SNP rs5219 (p.E23K) of *KCNJ11* and T2DM.

Num	Study ID	Type	Race / ethnicity	OR	95% C.I.	p	Case^*^	Control^*^	Fr.^§§^
1	U.K. cohort. Sakura (1996) [20]#	CCCG	Caucasian	1.53	0.99–2.38	0.06	100(38+45+17)	82(44+27+11)	0.30
2	Danish. Hansen (1997) [21]#	CCCG	Caucasian	1.41	0.86–2.33	0.18	58 (21+26+11)	75 (33+34+8)	0.23
3	U.K. cohort. Inoue (1997) [14]#	CCCG	Caucasian	1.10	0.76–1.60	0.62	172(72+78+22)	96(38+52+6)^§^	0.23
4	Utah. Inoue (1997) [14]#	CCCG	Caucasian	0.86	0.55–1.33	0.48	119 (52+55+12)	68 (21+44+3) ^§^	0.27
5	French. Hani (1998) [45]	CCCG	Caucasian	1.65	1.18–2.30	0.003	191 (53+87+51)	114 (45+53+16)	0.27
6	Japanese. Keiko (1999) [19] #	CCCG	Asian	1.12	0.62–2.04	0.71	31 (11+13+7)	76 (22+46+8) ^§^	0.41
7	U.K. cohort. Gloyn (2001) [22]	CCCG	Caucasian	1.30	1.04–1.63	0.02	360(133+161+66)	307(125+152+30)	0.25
8	Japanese. Yamada (2001) [49]	CCCG	Asian	1.22	0.78–1.90	0.54	103	73	0.34
9	North Zealand in Denmark. Nielsen (2003) [23]	CCCG	Caucasian	1.11	0.96–1.27	0.15	803 (287+382+134)	862 (330+408+124)	0.28
	**Meta-analysis 4, 5, 7, 9 by Nielsen et al. [23]**	**meta**	**Caucasian**	**1.49** ^**r**^	**1.20–1.83**	**0.002**	**1473 (525+685+263)**	**1351 (521+657+173)**	**0.27**
10	U.K. cohort. Gloyn (2003) [50]	CCCG	Caucasian	1.18	1.04–1.34	0.01	854 (308+412+134)	1182 (491+534+157)	0.26
	**Meta-analysis 1–5, 7 by Gloyn (2003) [50]**	**meta**	**Caucasian**	**1.30**	**1.13–1.49**	**0.0003**	**1000**	**742**	**ND**
	**Meta-analysis 1–5, 7, 10 by Gloyn (2003) [50]**	**meta**	**Caucasian**	**1.23**	**1.12–1.36**	**1.5 x 10** ^**–5**^	**1854**	**1924**	**ND**
11	U.K. cohort. Barroso (2003)[51]	CCCG	Caucasian	1.19	0.99–1.43	0.07	499 (198+220+81)	494 (212+225+57)	0.24
	**Meta-analysis 3, 5–9 by Florez et al. (2004) [48]**	**meta**	**Caucasian**	**1.14**	**1.06–1.22**	**0.0002**	**2879**	**3055**	**ND**
12	Scandinavian. Florez (2004) [48]	CCCG	Caucasian	1.19	1.00–1.43	0.05	477 (113+244+120)	473 (129+250+94)	0.46
13	Canadian. Florez (2004) [48]	CCCG	Caucasian	1.05	0.71–1.55	0.82	104 (27+54+23)	98 (27+50+21)	0.47
14	Sweden. Florez (2004) [48]	CCCG	Caucasian	1.23	1.03–1.48	0.02	496 (174+237+85)	506 (209+229+68)	0.36
	**Meta-analysis 12–14 and sibships by Florez et al. (2004) [48]**	**meta**	**Caucasian**	**1.17**	**1.05–1.32**	**0.003**	**1077**	**1077**	**ND**
	**Meta-analysis 3, 5, 7, 9–14 and sibships by Florez et al. (2004) [48]**	**meta**	**Caucasian**	**1.15**	**1.08–1.22**	**< 10** ^**–5**^	**5083**	**4747**	**ND**
15	Danish. Hansen (2005) [52]	CCCG	Caucasian	1.19	1.09–1.31	0.0002	1187 (423+568+196)	4791 (1955+2195+641)	0.36
16	Netherland. van Dam (2005) [53]	CCCG	Caucasian	1.27	0.98–1.65	0.07	192(66+92+34)	296 (119+141+36)	0.36
17	U.K. cohort. Weedon (2006) [54]	CCCG	Caucasian	1.14	1.05–1.23	0.001	2332	3592	0.35
18	Japanese. Yokoi (2006) [26]	CCCG	Asian	1.08	0.97–1.21	0.15	1590 (610+734+246)	1244 (503+570+171)	0.37
19	WTCCC. 2007 [55]	CCGWA	Caucasian	1.15	1.05–1.25	1.3 x 10^–3^	1924	2938	ND
20	Diabetes Genetics Initiative (DGI. 2007) [56]	CCGWA	Caucasian	1.15	1.09–1.21	1.0 x 10^–7^	6529	7252	0.47
21	Finland-United States Investigation of NIDDM Genetics (FUSION. 2007) [57]	CCGWA	Caucasian	1.11	1.02–1.20	0.014	2376	2432	0.46
	**Meta-analysis 19–21 by Saxena et al. (2007) [56]**	**meta**	**Caucasian**	**1.14**	**1.10–1.19**	**6.7 x 10** ^**–11**^	**10829**	**12622**	**ND**
22	Boston. Qi (2007) [58]	CCCG	Caucasian	1.25	1.09–1.44	0.002	714 (245+322+115)	1120 (446+505+127)	0.35
23	Japanese. Horikoshi (2007)[59]	CCCG	Asian	1.04	0.90–1.19	0.60	858 (334+393+131)	862 (332+417+113)	0.37
24	Korean. Koo (2007) [16]#	CCCG	Asian	1.28	1.10–1.49	0.002	761 (244+364+150)	630 (255+273+102) ^§^	0.38
25	Japanese. Sakamoto (2007) [60]	CCCG	Asian	1.21	1.05–1.38	0.007	906 (333+446+127)	889 (386+396+107)	0.34
26	Japanese. Doi (2007) [61]	PCG+CCCG	Asian	1.26	1.09–1.45	0.002	550 (202+263+85)	1433 (617+655+161)	0.34
27	Czech. Cejkova (2007) [62]	CCCG	Caucasian	1.01	0.71–1.43	0.96	172 (66+85+21)	113 (48+47+18)	0.37
28	African-American subject. Sale (2007) [17] #	CCCG	African-American	0.69^d^	0.49–0.99	0.045	577	596^§^	0.07
29	Finnish. Willer (2007) [63]	CCCG	Caucasian	1.22	1.08–1.38	0.002	1114 (284+560+270)	953 (286+486+181)	0.44
30	Japanese. Omori (2008) [64]	CCCG	Asian	1.25	1.08–1.46	0.003	1630	1064	0.36
31	D.E.S.I.R. cohort. Vaxillaire (2008) [65]	CCCG	Caucasian	1.09	0.91–1.29	0.36	327 (101+137+49)	2684 (994+1287+403)	0.39
32	MalmoPreventive Project (MPP). Lyssenko (2008)[39]	PCG	Caucasian	1.13	1.06–1.21	3.6 x 10^–4^	15600 (2063 with T2DM past 23.5 years)	13537	0.41
33	Botnia in Finland. Lyssenko(2008) [39]	PCG	Caucasian	0.98	0.75–1.26	0.85	2635 (138 with T2DM past 23.5 years)		0.51
34	Saudi. Alsmadi (2008) [47]	CCCG	Arab	1.69	1.30–2.20	0.00009	550 (341+187+22)	335(252+75+8)	0.14
35	Ashkenazi Jewish. Bronstein (2008) [18] #	CCCG	Ashkenazi Jewish	ND	ND	ND	1131	1147^§^	0.39
36	Sikh Diabetes Study. Sanghera (2008) [9]	CCCG	Indian	0.86	0.71–1.04	0.12	532 (226+247+59)	374 (148+169+57)	0.38
37	France and Switzerland. Cauchi (2008) [40]	CCCG	Caucasian	0.96	0.90–1.03	0.28	2734 (1112+1220+402) ^§^	4234 (1625+2006+603)	0.38
38	Japanese NIBI. Takeuchi (2009) [66]	CCGWA	Asian	1.07	1.01–1.13	0.01	5461 (2182+2511+768)	6894 (2883+3121+890)	0.36
	**Meta-analysis 23, 30 & 38 by Takeuchi et al (2009) [66]**	**meta**	**Japanese**	**1.09**	**1.04–1.13**	**3.4 x 10** ^**–4**^	**7954 (3129+3667+1158)**	**8809 (3638+4050+1121)**	**0.36**
39	Shanghai Diabetes Study. Hu (2009) [67]	CCCG	Asian	1.14	1.03–1.25	0.008	1849	1785	0.39
40	Japanese. Tabara (2009) [68]	CCCG	Asian	1.18	0.97–1.43	0.09	484 (169+232+83)	397 (152+195+50)	0.37
41	Chinese. Zhou (2009) [69]	CCCG	Asian	1.09	0.99–1.20	0.09	1848 (656+863+329)	1910 (692+930+288)	0.39
	**Meta-analysis of 8, 18, 24, 25, 26, 30, 40, 41 by Zhou et al (2009) [69]**	**meta**	**Asian**	**1.15**	**1.10–1.21**	**3 x 10** ^**–9**^	**7874**	**7629**	**ND**
42	Chinese Han population from Beijing. Wang (2009) [70]	CCCG	Asian	1.40	1.12–1.76	0.004	400	400	ND
43	Russian (Moscow). Chistakov (2009) [12]#	CCCG	Caucasian	1.54	1.08–2.20	0.023	127 (28+72+29)	117 (36+69+12) ^§^	0.40
44	Tunisian population. Ezzidi (2009) [71]	CCCG	Arab	1.15	0.97–1.36	0.12	805 (371+352+82)	503 (250+213+40)	0.29
45	USA. Cornelis (2009)[72]	CCCG	Caucasian	1.08	1.00–1.17	0.04	2709 (1055+1275+379)	3344 (1382+1536+426)	0.36
46	Ashkenazi Jewish. Neuman (2010) [7]	CCCG	Ashkenazi Jewish	1.05	0.90–1.23	0.52	573 (228+266+79)	843 (339+404+100)	0.36
47	Han Chinesse. Wen (2010)[73]	CCCG	Asian	1.07	0.95–1.21	0.26	1165 (395+587+183)	1135 (425+517+193)	0.40
48	Indo-European ethnicity. Chauhan (2010) [74]	CCCG	Indo-European	1.39	1.26–1.54	6.7 x 10^–11^	2486	2678	0.31
49	Russian (Moscow). Chistakov (2010) [15]#	CCCG	Caucasian	1.41	1.20–1.66	0.00003	588 (134+339+115) ^§^	597 (183+352+62) ^§^	0.40
50	Chinese. Liu (2010)[75]	CCCG	Asian	1.26	1.03–1.55	0.02	397 (131+180+86)	392 (147+187+58)	0.39
51	UK Asian Diabetes Study (UKADS) and DiabetesGenetics in Pakistan (DGP). Rees (2011) [41]	CCCG	Asian	0.98	0.88–1.08	0.71	1678 [857(UKADS) 821(DGP)]	1584 [417(UKADS) 1167 (DGP)]	0.38
52	Indo-European ethnicity. Chavali (2011) [10]	CCCG	Indo-European	1.00	0.88–1.13	0.89	1019	1006	0.35
	**Meta-analysis of 30. 38 by Yang et al (2012) [76]**	**meta**	**Japanese**	**1.23**	**1.13–1.35**	**2 x 10** ^**–6**^	**7091**	**7958**	**ND**
	**Meta-analysis of 39. 41. 42. 47 by Yang et al (2012)[76]**	**meta**	**Chinese**	**1.12**	**1.06–1.19**	**5 x 10** ^**–5**^	**5262**	**5231**	**ND**
	**Meta-analysis of 24. 30. 38. 39. 41. 42. 47 by Yang et al (2012) [76]**	**meta**	**Asian**	**1.16**	**1.11–1.21**	**4 x 10** ^**–11**^	**13114**	**13819**	**ND**
53	Tunisians. Mtiraoui (2012) [77]	CCCG	Arab	1.27	1.09–1.47	8 x 10^–4^	1470	838	ND
54	Mongolian. Odgerel (2012) [8]	CCCG	Mongolian	1.07	0.80–1.44	0.65	177	216	0.32
	**Meta-analysis of 49 studies by Gong [78]**	**meta**	**All**	**1.13**	**1.10–1.15**	**7 x 10** ^**–8**^	**64403**	**122945**	**ND**
55	urban Ghana. Danquah (2013)[79]#	CCCG	Akan	NA	NA	NA	675(674+1+0)	377 (377+0+0)	0.00
	**Meta-analysis of 15 European studies by Qin (2013) [80]**	**meta**	**European**	**1.16**	**1.11–1.20**	**4 x 10** ^**–11**^	**9165**	**13300**	**36.8**
	**Meta-analysis of 13 Asian studies by Qin (2013) [80]**	**meta**	**Asian**	**1.11**	**1.04–1.20**	**0.002**	**13213**	**13229**	**ND**
	**Meta-analysis of 7 Chinese studies by Qin (2013) [80]**	**meta**	**Chinese**	**ND**	**ND**	**0.28**	**6308**	**6213**	**0.40**
	**Meta-analysis of 5 Japanese studies by Qin (2013) [80]**	**meta**	**Japanese**	**1.16**	**1.08–1.24**	**5 x 10** ^**–5**^	**3561**	**4039**	**0.35**
	**Meta-analysis of 33 studies by Qin (2013) [80]**	**meta**	**All**	**1.15**	**1.10–1.21**	**7 x 10** ^**–7**^	**23262**	**27042**	**ND**
	**Meta-analysis of 22 studies by Qiu (2014)[42]**	**meta**	**Caucasian**	**1.12**	**1.08–1.16**	**10** ^**–9**^	**ND**	**ND**	**0.40**
	**Meta-analysis of 14 studies by Qiu (2014) [42]**	**meta**	**East Asian**	**1.13**	**1.08–1.17**	**10** ^**–7**^	**ND**	**ND**	**0.36**
	**Meta-analysis of 5 studies by Qiu (2014) [42]**	**meta**	**Indians**	**1.06**	**0.87–1.29**	**0.56**	**ND**	**ND**	**0.34**
	**Meta-analysis of 7 studies by Qiu (2014) [42]**	**meta**	**Others**	**1.09**	**0.97–1.23**	**0.15**	**ND**	**ND**	**ND**
	**Meta-analysis of 48 studies by Qiu (2014) [42]**	**meta**	**All**	**1.12**	**1.09–1.16**	**3 x 10** ^**–16**^	**ND**	**ND**	**ND**

Results of previous meta-analysis are shown in bold.

* Total number persons in groups is given. Number of people with a particular genotype is shown in brackets. EE+EK+KK respectively.

^§^—not in Hardy-Weinberg Equilibrium.

^§§—^frequency of T allele (p.23K) in the control group.

#—data was excluded from the meta-analysis (see study selection);

Abbreviations: ^r^- under recessive model; ^d^—dominant model; additive model is default variant; ND—no available data; CCGWA—genome-wide association study. Case-control design; CCCG—case-control design candidate gene study; meta—meta-analysis; PCG-prospective candidate gene study.

#### Meta-analysis results

**Fig 1:**
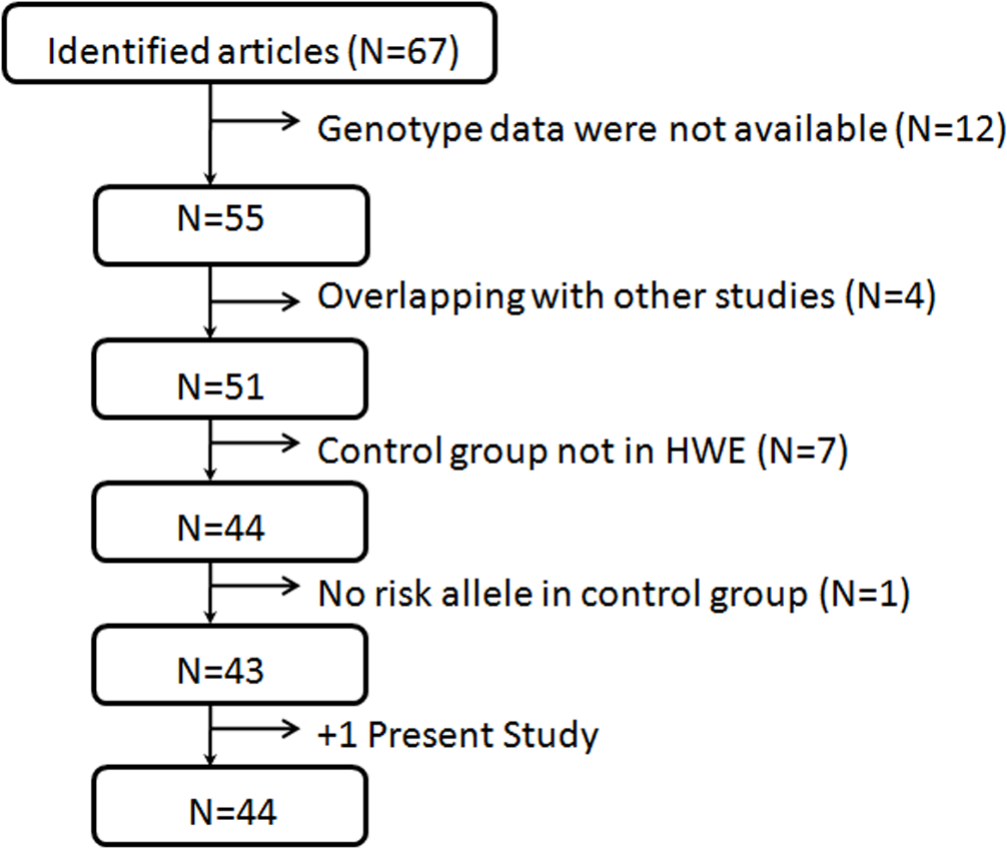
Study selection for meta-analysis of rs5219 of KCNJ11.

We identified a total of 67 potential articles for meta-analysis, but 24 were excluded for not meeting the required criteria (Fig 1). We did not include the Utahand U.K. samples from Inoue et al. [14] because p.E23K failed to meet HWE in the control group (p = 0.002 and p = 0.04, respectively). Results from both studies with Russian samples were excluded because in one study, rs5219 failed to obey HWE in T2DM (p = 0.0002) and control (p = 2 x 10^–8^) groups, and in the other study the control group failed to meet this criterion (p = 0.02) [12,15]. Results from Koo et al. [16] were excluded because control group genotypes reached borderline significance for HWE (p = 0.05), and the results of Sale et al. [17] were also excluded because rs5219 (p = 0.0009) deviated from HWE proportions in African-American patients. In that study, the minor allele for this SNP was rare (0.056) in the population, and results for only dominant models were available. The results from Bronstein et al. [18] also exhibited a significant deviation (P < 0.05) from HWE, and the results of Keiko et al. [19] were also excluded due to a HWE mismatch. We did not include data comprising 182 and 268 British patients studied by Sakura et al. [20] and Inoue et al. [14], respectively, as well as 133 Danish patients reported by Hansen et al. [21]. These were included in the UKPDS cohort [22] and Nielsen samples were addressed in Nielsen et al.[23].Results of Miyake et al. [24] were excluded because they overlapped with the patients of Horikoshi et al. [25] and Yokoi et al. [26]. Studies by Altshuler et al.[27], Love-Gregory et al. [28], Sladek et al. [29], Steinthorsdottir et al. [30], Salonen et al. [31], Hanson et al. [32], Hayes et al. [33], Rampersaud et al. [34], Yu et al. [35], Turki et al. [36], Thorsby et al. [37], and Yamauchi et al. [38] were also excluded from our meta-analysis because genotype data were not available.

**Fig 2:**
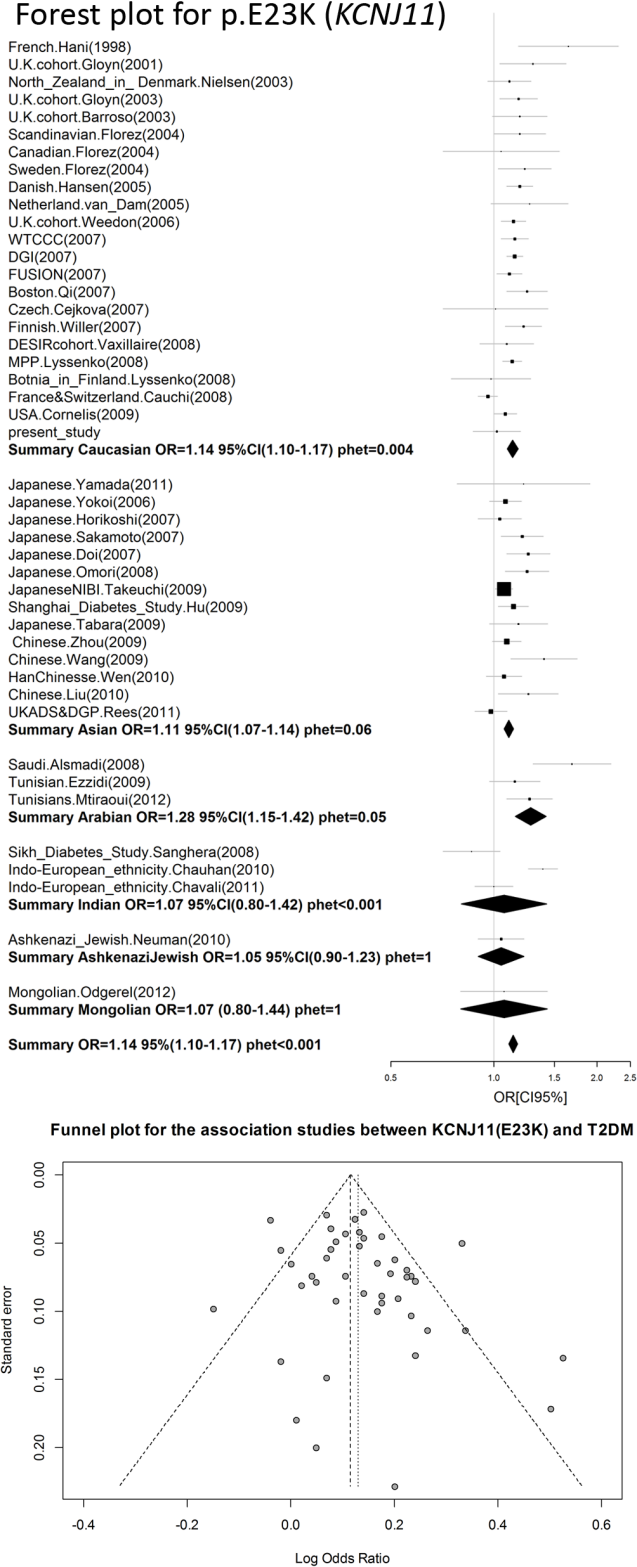
Forest and funnel plots of meta-analysis of rs5219 of KCNJ11.

**Fig 3:**
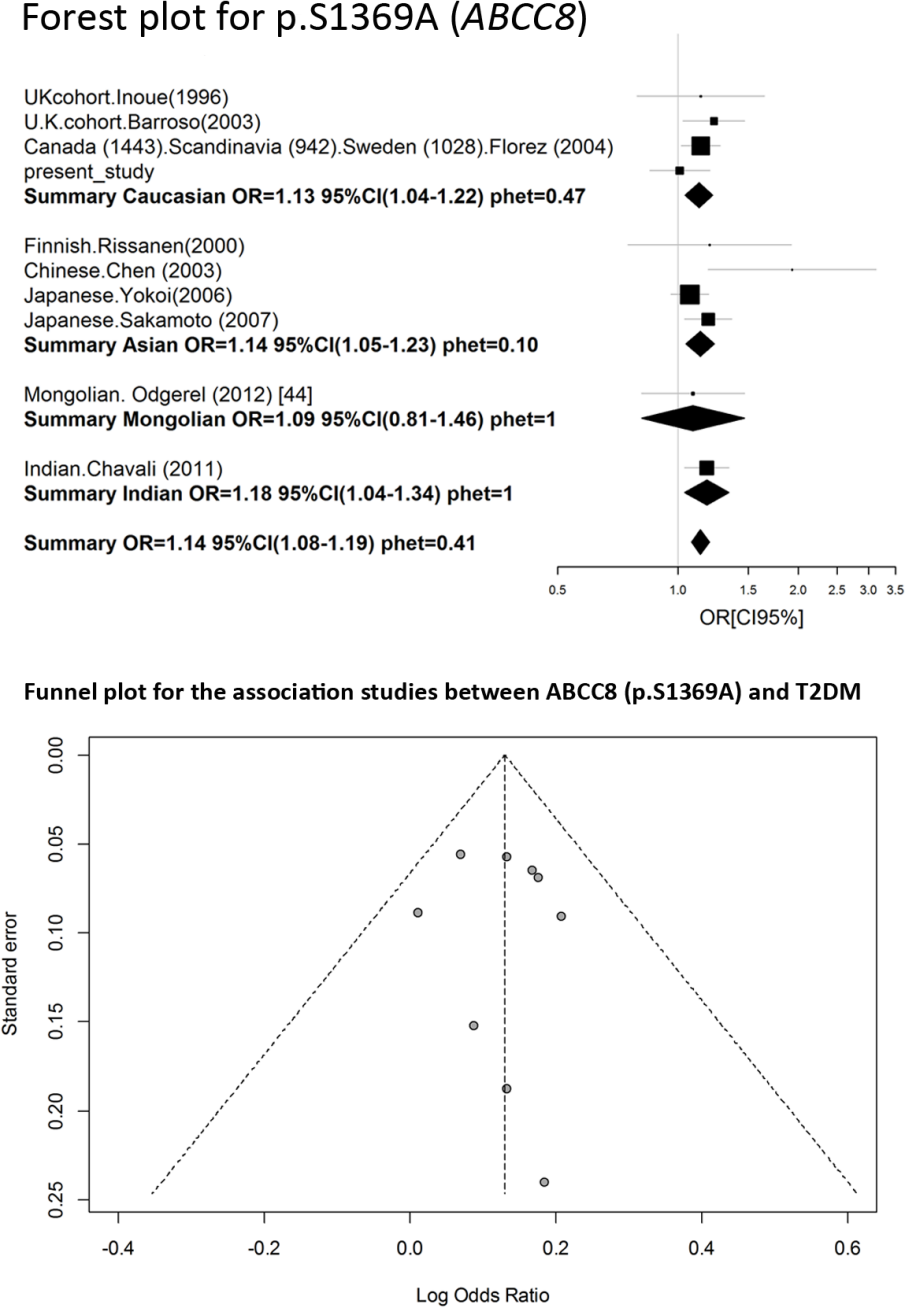
L’Abbe plot for KCNJ11 (p.E23K).

**Fig 4:**
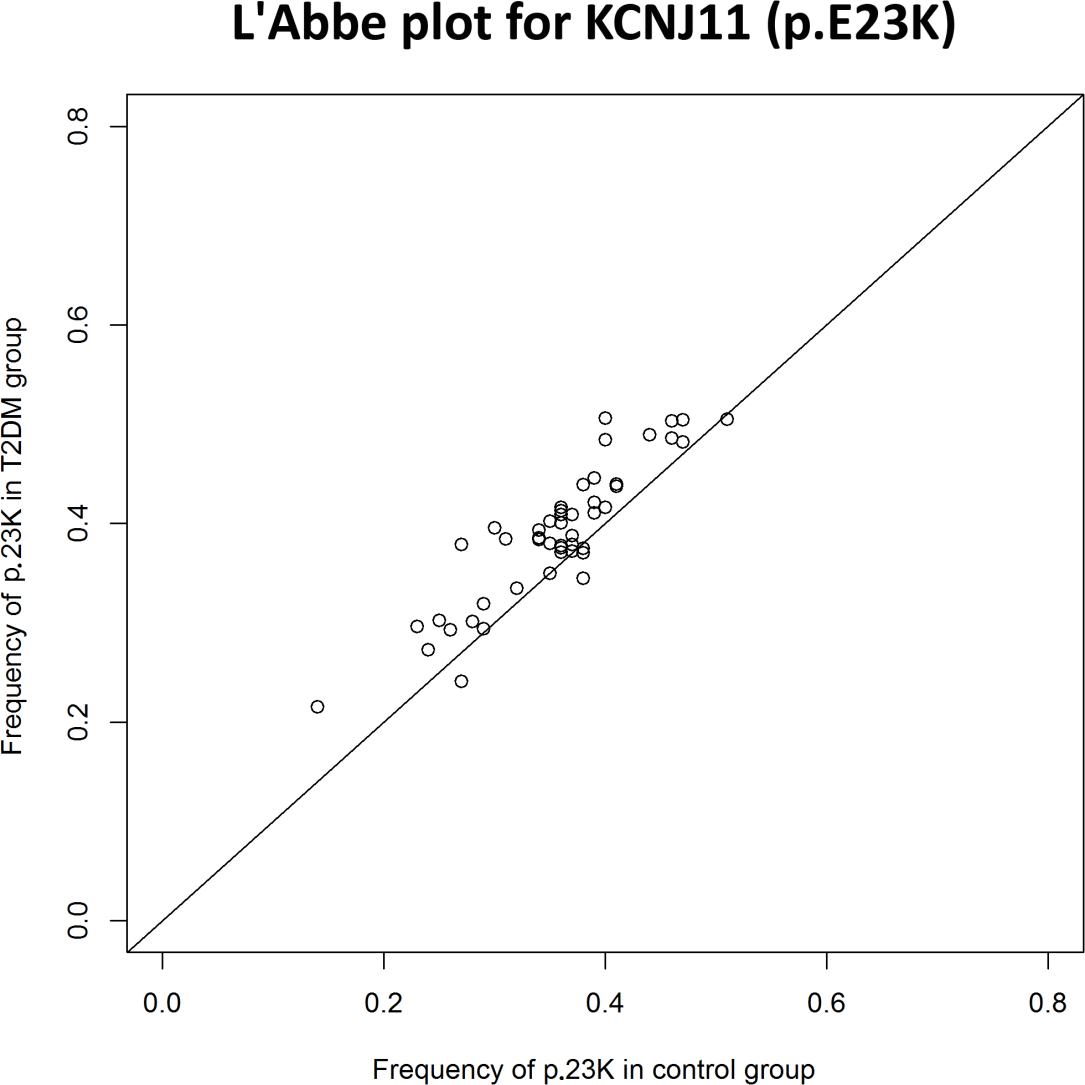
OR dependence on the effective sample size.

**Fig 5:**
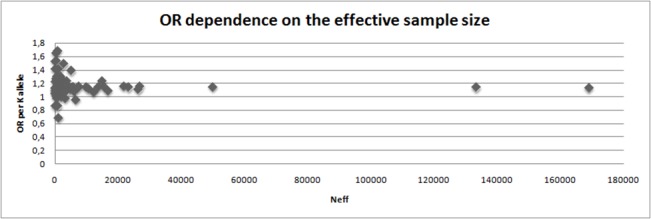
P-value dependence on the effective sample size.

We performed a meta-analysis of our own results combined with previously published data on the association between rs5219 and T2DM (Table 4, Fig 2). A total of 56,210 T2DM patients and 81,088 control patients from 44 studies were included (total sample size was 137,298). In 41 studies, the 5219[T] allele was the risk allele; in four studies, the opposite allele was identified as the risk [9,39–41]. In the first meta-analysis, the total OR for all studies was 1.14 (95% CI: 1.11–1.17) with a statistical significance of *P* = 6 x 10^–22^, and the heterogeneity test revealed significant differences between studies (*P*< 0.001).No publication bias for the SNP rs5219 was found according to Begg’s correlation analysis (corrected z = 1.26 and corrected P = 0.21); however, according to the Egger there was publication bias between studies test (t = 1.90, p = 0.06) (Fig 2). We then divided all studies into six groups according to ethnicity (Caucasian, Asian, Indian, Arabian, Mongolian, and Ashkenazi Jewish) and conducted a meta-analysis for each group. Significant heterogeneity was found for the Caucasian, Indian, and Arabian subgroups. The highest OR was obtained for Arabians [OR = 1.28, 95%CI (1.15–1.42)] and the lowest OR was observed for Asians [OR = 1.11, 95%CI (1.07–1.14)].We found that rs5219 was not associated with T2DM for Indian, Mongolian, and Ashkenazi Jewish groups. We next performed a meta-analysis that included all seven studies in which the control group did not obey the HWE (S1 Fig, Table 4) [12,14–16,19–21]. Sample sizes of the T2DM and control groups increased to 57,994 and 82,733, respectively, with a total sample size of 140,727. The pooled OR was 1.15 [95% CI (1.12–1.19)] with significant heterogeneity between studies (p < 0.001).Previous meta-analyses also exhibited heterogeneity between studies [42]due to the following possible reasons: ethnicity, sample size, mean age of cases and controls, gender distribution in cases and controls, and body mass index (BMI). However, BMI alone only explained approximately 11% of the heterogeneity (p = 0.03).We plotted an L’Abbe plot [43]to visualize differences in the frequency of rs5219 between populations (Fig 3) and assessed the dependence of ORs and p values from effective sample sizes (Figs 4 and 5). We used the effective population size as given by the doubled harmonic means of case and control group sizes to overcome the differences in proportion between controls and cases.

**Table 4:** Results of meta-analysis.

Group	Studies	Case	Control	OR (95%CI)	p-value	phet
**Results of meta-analysis of p.E23K (*KCNJ11*)**						
Caucasian	23	29679	54233	1.14 (1.10–1.17)	6 x 10^–13^	0.004
Asian	14	18919	20062	1.11 (1.07–1.14)	3 x 10^–8^	0.06
Indian	3	4037	4058	1.07 (0.80–1.42)	0.65	<0.001
Arabian	3	2825	1676	1.28 (1.15–1.42)	6 x 10^–6^	0.02
Ashkenazi Jewish	1	573	843	1.05 (0.90–1.23)	0.52	1
Mongolian	1	177	216	1.07 (0.80–1.44)	0.65	1
Total (in HWE)	44	56 210	81 088	1.14 (1.11–1.17)	6 x 10^–22^	<0.001
Total (+ not in HWE)	52	57 994	82 733	1.15 (1.12–1.19)	4 x 10^–25^	<0.001
**Results of meta-analysis of p.S1369A (*ABCC8*)**						
Caucasian	4	3794	2725	1.13 (1.04–1.22)	0.004	0.47
Asian	4	2336	2908	1.14 (1.05–1.23)	0.002	0.10
Indian	1	1019	1006	1.18 (1.04–1.34)	0.01	1
Mongolian	1	177	216	1.09 (0.81–1.46)	0.58	1
Total (in HWE)	10	5897	6441	1.14 (1.08–1.19)	2 x 10^–6^	0.41

phet—p-value of heterogeneity between studies

### Meta-analysis of rs757110 of *ABCC8*

**Fig 6:**
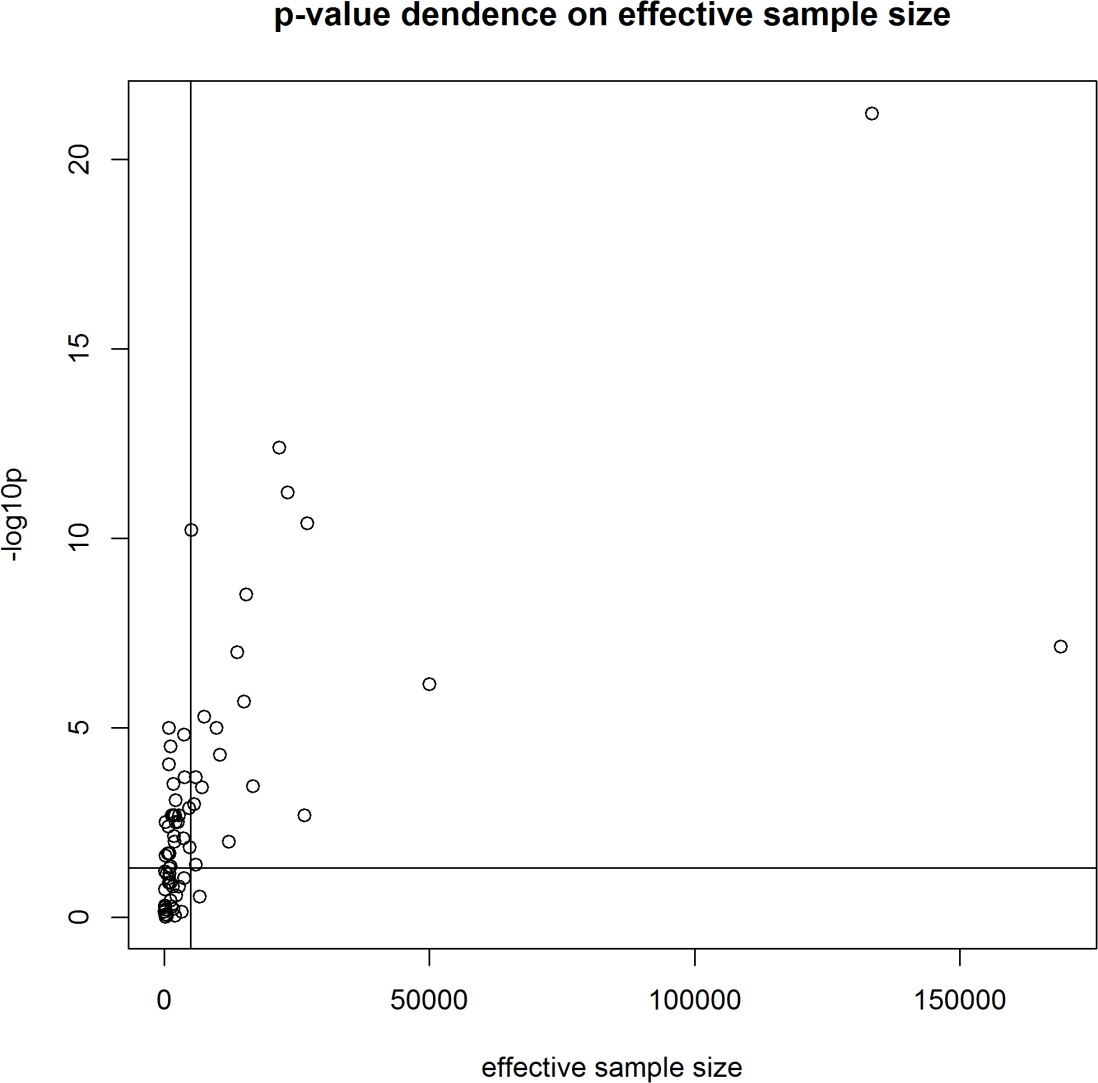
Forest and funnel plots of meta-analysis of rs757110 of ABCC8.

A search for publications on the rs757110 was conducted in a similar manner as described for the rs5219 search. All of the available information identified is shown in Table 5. We exclude data of both Hani et al. and Ishiyama-Shigemoto et al. [44] that were referenced by Qin et al. We found no information in the reference manuscript [45], and some data in the Qin manuscript were incorrect. Data from Ohta et al. [46] were also excluded because the original article did not contain sufficient information. We did not include the Utah patients from Inoue et al. [14] because p.S1369A did not comply with HWE for the control group (p = 0.01).The Begg’s correlation analysis (corrected z = 1.34 and corrected P = 0.18) and the Egger test (t = 1.25, p = 0.25) found no publication bias for SNP rs5219 (Fig 6). A meta-analysis was conducted on a total of 10 studies, including our present study (Fig 5, Table 4). The total sample size was 14,136, which included 7281 cases and 6855 controls. The pooled OR was 1.14 [95% CI (1.08–1.19); p = 2 x 10^–6^] and there was no heterogeneity between studies (p = 0.41).

**Table 5:** Characteristics studies of association SNP rs757110 (p.S1396A) of *ABCC8* and T2DM.

Num	Study ID	Type	Race/Ethnicity	OR	95% C.I.	p	Case^§§§^	Control^§§§^	Fr.^§§^
1	Utah. Inoue (1996) [81] #	CCCG	Caucasian	0.96	0.66–1.41	0.85	133 (58+58+17)	103 (37+59+7)^§^	0.35
2	UK cohort. Inoue (1996) [81]	CCCG	Caucasian	1.14	0.79–1.63	0.49	187 (98+67+22)	120 (64+47+9)	0.27
3	French. Hani (1998)* #	CCCG	Caucasian	ND	ND	ND	168	106	0.34
4	Japanese. Ohta (1998) # [46]	CCCG	Asian	ND	ND	NS	100 (46+36+18)	67	ND
5	Japanese. Ishiyama (1998)* # [44]	CCCG	Asian	1.10	0.85–1.43	ND	1590 (570+744+276)	1244 (463+587+194)	0.39
6	Finnish. Rissanen (2000) [82]	CCCG	Asian	1.20	0.75–1.90	0.45	40	377	0.42
7	U.K. cohort. Barroso (2003) [51]	CCCG	Caucasian	1.23	1.03–1.48	0.02	502 (189+225+88)	499 (205+238+56)	0.35
8	Chinese. Chen (2003)*	CCCG	Asian	1.93	1.19–3.14	0.008	105 (20+60+25)	51 (19+27+5)	0.36
9	Canada (1443), Scandinavia (942), Sweden (1028). Florez (2004) [48]	CCCG	Caucasian	1.14	1.02–1.28	0.02	1721	1692	ND
10	Japanese. Yokoi (2006) [26]	CCCG	Asian	1.07	0.96–1.19	0.23	1244 (463+587+194)	1590 (570+744+276)	0.41
11	Japanese. Sakamoto (2007)* [60]	CCCG	Asian	1.19	1.04–1.36	0.01	902 (310+441+151)	890 (357+407+126)	0.37
12	Indian. Chavali (2011)* [10]	CCCG	Indian	1.18	1.04–1.34	0.01	1019	1006	0.38
13	Mongolian. Odgerel (2012) [8]	CCCG	Mongolian	1.09	0.81–1.46	0.58	177	216	0.32
	**Meta-analysis of 9studies by Qin (2013) [76]**	**meta**	**All**	**1.12**	**1.07–1.18**	**10** ^**–6**^	**5835**	**5261**	**ND**

Results of meta-analysis are shown in bold.

^§^—not in Hardy-Weinberg Equilibrium.

^§§—^frequency of G allele (p.1369G) in the control group.

§§§Total number persons in groups is given. Number of people with a particular genotype is shown in brackets. SS+SA+AA, respectively.

#—data was excluded from the meta-analysis (see text in article).

Abbreviations: ND—no available data; CCGWA—genome-wide association study. case-control design; CCCG—case-control design candidate gene study; NS—not significant; meta—meta-analysis.

*—data are given according to article Qin et al. [80]

We also performed a meta-analysis on the Caucasian, Asian, Indian, and Mongolian ethnic groups, but the Indian and Mongolian groups were presented in one study. The pooled OR was 1.13 (1.04–1.22) for Caucasians and 1.14 (1.05–1.23) for Asians.

### Linkage disequilibrium between rs757110 (*ABCC8* p.S1369A) and rs5219 (*KCNJ11* p.E23K) and association of haplotypes

We estimated the linkage disequilibrium (LD) between SNPs rs5219 and rs757110 and found that they were in LD (D’ = 0.766, r^2^ = 0.5633, χ^2^ = 1012.81). We also performed a haplotype analysis for rs5219 andrs757110 and found that the haplotype rs757110[T]-rs5219[C] was associated with T2DM [Table 6; OR = 1.52, 95% CI (1.04–2.24); empirical p = 0.03].

**Table 6:** Analysis of association between T2DM and haplotypes at SNPs rs5219 and rs757110.

Rs757110	Rs5219	Frequency in Cases	Frequency in Control	Sample frequency	OR	95% C.I.	Empirical p-value
T	C	0.555842	0.5777821	0.561038	reference	—	—
T	T*	0.059404	0.036913	0.054090	***1*.*52***	***1*.*04–2*.*24***	***0*.*03***
G*	C	0.067713	0.050198	0.063545	1.27	0.93–1.73	0.14
G*	T*	0.317041	0.335067	0.321327	0.99	0.83–1.17	0.88

Abbreviations: 95% CI, 95% confidence interval; OR, odds ratio; Sample frequency—haplotype frequency in MS and control groups together; empirical p-value—p-value of association haplotype with MS;

*—marked the risk allele from meta-analysis.

Analysis was performed using logistic regression.

## Discussion

This study assessed whether SNPs rs5219 and rs757110 are associated with a predisposition to T2DM in Siberians and found that neither SNP was associated with the disease. The power of the study reached 28% for both SNPs, and approximately 2400 case-control pairs would be required for 80% power to detect the risk allele among Caucasians.

We also reviewed all available previous studies involving p.E23K (*KCNJ11*) and p.S1369A (*ABCC8*) (Tables 3 and 5). To date, 67 original studies and 10 meta-analyses have analyzed the association of the p.E23K variant of *KCNJ11* with T2DM (Fig 1, Table 3). Cohorts of American, Danish, French, Japanese, North Zealand, Scandinavian, Canadian, Sweden, Dutch, Finland, Boston cohort, Korean, Czech, African-American, Finish, Ashkenazi Jewish, Indians, Chinese, Arabian, Mongolian, urban Ghana, and Russian patients have been studied. The OR for the p.23K allele ranged from 0.69 in African Americans [17] to 1.69 in Arabians [47]. The first pooled OR from meta-analysis was 1.49 [23] and became 1.11–1.14 when the effective sample size reached 20,000 people (Fig 4). A similar trend in the OR was observed in original studies of Asians and Caucasians. In our meta-analysis of p.E23K, the pooled OR was 1.14 and 1.11 for the Caucasian and Asian cohorts, respectively (Table 4). The frequency of the K allele ranged from 0% in urban Ghana patients to 50% in Finnish patients in previous studies. Moreover, the K allele frequency was different between the race controls: 0.40 (95% CI: 0.37–0.42) for Caucasian, 0.36(95% CI: 0.34–0.38) for Asian, and 0.34(95% CI: 0.27–0.41) for Indian [42]. Schwanstecher et al. hypothesizedthat a high frequency of polymorphisms may be a consequence of the selective advantages. Reducing glucose uptake by muscle and fat led to better glucose uptake by insulin-independent tissues, such as brain [6].We also analyzed the dependence of OR on the risk allele (p.23K) frequency using an L’abbe plot (Fig 3) and found that the OR depends more on the effective sample size than the frequency of the risk allele in the population. We also assessed if the p-values from all previous studies depended on effective sample sizes (Fig 5). The vertical line corresponds to an effective sample size of 5000, which is necessary for a predictive power of 80%. The horizontal line corresponds to p = 0.05. It is obvious that for all studies except one in which the sample size was more than 5000, the effective sample size showed statistically significant differences in p.E23K between the control and T2DM patients. However, all of the meta-analyses conducted had problems of heterogeneity between studies, and heterogeneity remained after separate analyses of individual races, including Caucasian, Indian, and Arabian populations. Only Asians trended towards homogeneity. Qiu et al. reported BMI as the reason for heterogeneity, which accounted for approximately 11% of the heterogeneity among the individuals studied (p = 0.03) [42].We hypothesize that heterogeneity may also be caused by the younger mean age of the control group, since T2DM is a disease with a relatively late onset in life. Thus, a young control group may include patients that will develop T2DM in the future. The number of such persons in the control group depends on the prevalence of T2DM in the cohort set, which in turn depends on various external factors. The higher the proportion of futureT2DM patients in the control, the more the true value OR is understated.

The association between p.S1369A (*ABCC8*) has been reported in nine available studies to date, and the pooled OR was 1.14 [95% CI (1.08–1.19)]. Heterogeneity between studies was absent. In contrast to p.E23K, p.S1369Awasassociated with T2DM not only in Asians and Caucasians, but also in Indians (Table 6, Fig 6), where the same OR ranged from 1.13 to 1.18. The frequency of the p.1369A allele only modestly varied between populations (27–42%).

In summary, our data support the hypothesis that genetic variation located in the *ABCC8/KCNJ11* region is associated with the development of T2DM with an OR of approximately 1.15. We also found that haplotype rs757110[T]-rs5219[C] (p.23K/p.S1369) was associated with T2DM [OR = 1.52, 95% CI (1.04–2.24); empirical p = 0.03). In a previous study of *ABCC8/KCNJ11*, the region haplotype (p.23K/p.1369A) was also associated with T2DM [OR = 1.15 95% CI (1.03–1.29); p = 0.01] [48]. In fact, that study could not distinguish the p.23K and p.1369A alleles because the LD between SUR1 p.S1369A and Kir6.2 p.E23K was very strong in the cohort (r^2^ = 0.9). Moreover, each chromosome containing the K allele in p.E23K also contained the A allele in p.S1369A (frequency of p.E23/p.S1369 = 0.57, p.23K/p.1369A = 0.42, and p.23K/p.S1369 = 0.01). In our study, the LD was not as strong (r^2^ = 0.5633), and the rare haplotype frequency was higher (p.E23/p.S1369 = 0.56, p.23K/p.1369A = 0.32, p.23K/p.S1369 = 0.06, and p.E23/p.1369A = 0.05). Based on our results, the p.23K/p.S1369 haplotype may be a T2DM causal variant. However, the association of p.23K/p.S1369 had borderline statistical significance, which was most likely due to the small sample size. Further studies are needed in much larger sample sizes and in populations in which chromosomes recombinant for p.E23K and p.S1369Aare higher than in European populations [48]. Given the low frequency of p.23K/p.S1369 and p.E23/p.1369A haplotypes in Europeans (~1%) and the current OR of 1.15, approximately 120,000 case/control pairs are necessary to distinguish between the two [23].

## Conclusions

Our calculations identified causal genetic variation within the *ABCC8/KCNJ11* region for T2DM with an OR of approximately 1.15 in Caucasians and Asians. The OR value was not dependent on the frequency of p.E23K or p.S1369A in the populations.

## Supporting Information

S1 Fig Forest-pots of meta-analysis of rs5219 of KCNJ11 including all seven studies in which control group was not obeyed Hardy-Weinberg equilibrium. (TIF)

S1 Table PRISMA Checklist of items included in our meta-analysis. The table was formed in accordance with the requirements of the site http://www.prisma-statement.org/statement.htm.
(DOCX)
