# Supplementary figures and images for: Replication of KCNJ11 (p.E23K) and ABCC8 (p.S1369A) Association in Russian Diabetes Mellitus 2 Type Cohort and Meta-Analysis
Source: PLoS One. 2015 May 8;10(5):e0124662. doi: 10.1371/journal.pone.0124662 (PMC4425644; doi:10.1371/journal.pone.0124662)

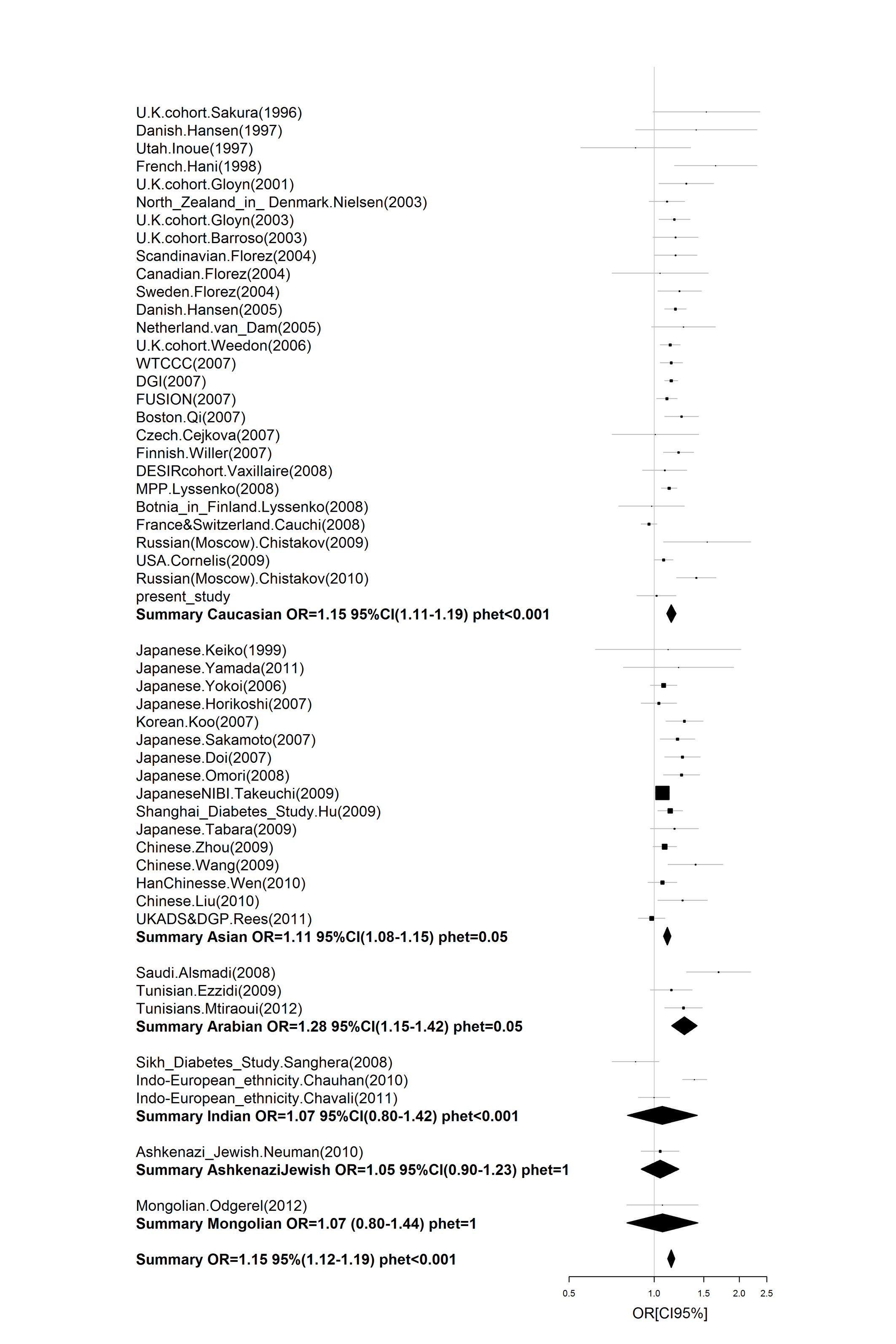

Supplement: S1 Fig — (TIF) [file pone.0124662.s001.tif]
